# Serum and Fecal Markers of Intestinal Inflammation and Intestinal Barrier Permeability Are Elevated in Parkinson’s Disease

**DOI:** 10.3389/fnins.2021.689723

**Published:** 2021-06-18

**Authors:** Laura Dumitrescu, Daciana Marta, Adela Dănău, Antonia Lefter, Delia Tulbă, Liviu Cozma, Emilia Manole, Mihaela Gherghiceanu, Laura Cristina Ceafalan, Bogdan Ovidiu Popescu

**Affiliations:** ^1^Department of Clinical Neurosciences, Neurology Division at Colentina Clinical Hospital, Carol Davila University of Medicine and Pharmacy, Bucharest, Romania; ^2^Department of Neurology, Colentina Clinical Hospital, Bucharest, Romania; ^3^Laboratory of Cell Biology, Neurosciences and Experimental Myology, “Victor Babeş”, National Institute of Pathology, Bucharest, Romania; ^4^Pathology Department, Colentina Clinical Hospital, Bucharest, Romania; ^5^Department of Cellular and Molecular Biology and Histology, Carol Davila University of Medicine and Pharmacy, Bucharest, Romania

**Keywords:** calprotectin, Parkinson’s disease, intestinal inflammation, intestinal barrier permeability, zonulin

## Abstract

Parkinson’s disease (PD) is characterized by alpha-synuclein misfolding with subsequent intraneuronal amyloid formation and accumulation, low grade neuroinflammatory changes, and selective neurodegeneration. Available evidence suggests that the pathology usually begins in the gut and olfactory mucosa, spreading to the brain via the vagus and olfactory nerves, by a prion-like mechanism. A causal relationship has not been established, but gut dysbiosis is prevalent in PD and may lead to intestinal inflammation and barrier dysfunction. Additionally, epidemiological data indicate a link between inflammatory bowel diseases and PD. Calprotectin and zonulin are markers of intestinal inflammation and barrier permeability, respectively. We evaluated their serum and fecal levels in 22 patients with sporadic PD and 16 unmatched healthy controls. Mean calprotectin was higher in PD, both in serum (14.26 mcg/ml ± 4.50 vs. 5.94 mcg/ml ± 3.80, *p* = 0.0125) and stool (164.54 mcg/g ± 54.19 vs. 56.19 mcg/g ± 35.88, *p* = 0.0048). Mean zonulin was also higher in PD serum (26.69 ng/ml ± 3.55 vs. 19.43 ng/ml ± 2.56, *p* = 0.0046) and stool (100.19 ng/ml ± 28.25 vs. 37.3 ng/ml ± 13.26, *p* = 0.0012). Calprotectin was above the upper reference limit in 19 PD serums and 6 controls (OR = 10.56, 95% CI = 2.17–51.42, *p* = 0.0025) and in 20 PD stool samples and 4 controls (OR = 30, 95% CI = 4.75–189.30, *p* = 0.000045). Increased zonulin was found only in the stool samples of 8 PD patients. Despite the small sample size, our findings are robust, complementing and supporting other recently published results. The relation between serum and fecal calprotectin and zonulin levels and sporadic PD warrants further investigation in larger cohorts.

## Introduction

Parkinson’s disease (PD) is an incurable disorder affecting more than 6 million people worldwide. Its incidence and prevalence increase with age, culminating in the eighth decade (Collaborators, 2018). Disability is related both to motor (i.e., parkinsonism) and non-motor symptoms (including hyposmia and constipation), slowly progressing over the course of many years (Postuma et al., 2015; Balestrino and Schapira, 2020).

The neuropathology of PD is defined by intraneuronal accumulation of alpha-synuclein amyloids, namely Lewy bodies and Lewy neurites, low grade neuroinflammation and selective neuronal dysfunction with subsequent neurodegeneration, involving mainly the aminergic neurocircuits (Braak et al., 2003a; Balestrino and Schapira, 2020). The development of sporadic PD is linked to several environmental or acquired factors that are thought to initiate and promote the disease, especially in genetically susceptible individuals (Hawkes et al., 2007, 2009; Balestrino and Schapira, 2020). The upstream pathogenic events that trigger the initial amyloid transformation of alpha-synuclein (i.e., misfolding, self-aggregation and cross-seeding of the conformational changes) are largely unknown. Increasing evidence suggests that the pathology may begin in the gut and/or the olfactory mucosa, spreading to the cortex by a prion-like mechanism (Braak et al., 2003a, b, 2006; Halliday et al., 2006; Hawkes et al., 2007, 2009; Masuda-Suzukake et al., 2013; Hilton et al., 2014; Svensson et al., 2015; Rietdijk et al., 2017). Intestinal inflammation and barrier dysfunction, as well as products of the gut microbiota (that may cause “leaky gut” and/or local inflammation and may trigger or enhance amyloidogenesis), are thus considered potential key players in the etiopathogenesis of PD (Devos et al., 2013; Houser et al., 2018; Schwiertz et al., 2018; Becker et al., 2019; Mulak et al., 2019; Rolli-Derkinderen et al., 2020; Romano et al., 2021).

Gut dysbiosis and inflammatory bowel disease (IBD) are associated with increased risk of PD (Hill-Burns et al., 2017; Minato et al., 2017; Camacho-Soto et al., 2018b; Heinzel et al., 2019; Park et al., 2019; Pietrucci et al., 2019; Villumsen et al., 2019; Weimers et al., 2019; Zhu et al., 2019; Baldini et al., 2020; Nishiwaki et al., 2020; Nuzum et al., 2020; Lee et al., 2021). Recent studies found that fecal markers of intestinal inflammation, such as fecal calprotectin (routinely used for diagnosing and monitoring IBD) and possibly markers of intestinal barrier permeability, such as fecal zonulin, are elevated in people with PD (Schwiertz et al., 2018; Mulak et al., 2019). Inflammatory shifts of stool immune profiles comparable to those seen in IBD, were also described in PD (Houser et al., 2018). Here we present our preliminary results on the association between sporadic PD and serum and fecal levels of calprotectin and zonulin. To the best of our knowledge, this is the first study evaluating serum calprotectin and zonulin in PD, previous studies assessing only their fecal levels.

## Materials and Methods

We performed a case-control study investigating the serum and fecal levels of calprotectin and zonulin in adult people with and without PD. Participants were recruited at Colentina Clinical Hospital (Bucharest, Romania) starting from April 2019. Study enrollment was based on predefined inclusion and exclusion criteria—see below. The protocol of the study was approved by the local Ethics Committee (EMI-BPs, 3/16.04.2019). Written informed consent, compliant with the Declaration of Helsinki and the European General Data Protection Regulation 2016/679, was obtained from all participants prior to study enrollment.

To be included in the sporadic PD group, patients had to fulfill the Movement Disorder Society (MDS) Clinical Diagnostic Criteria for either clinically established or clinically probable PD (Postuma et al., 2015); additionally, the patients must have had the onset of their motor symptoms after the age of 50 years, no autosomal dominant or recessive family history of PD and no other elements indicative for monogenic PD. The following exclusion criteria were applied: recent (less than 6 months prior to study sample collection) or concurrent gastrointestinal or systemic conditions, including infections or surgical interventions, or severe disability that may interfere with the results of the tests or preclude the clinical evaluation; antibiotic treatment within the past 3 months; and use within the past month of other drugs or supplements that may interfere with the results of the testing, such as steroidal or non-steroidal anti-inflammatory drugs (NSAIDs), including daily aspirin above 100 mg, and daily use of proton pump inhibitors (PPIs). Further exclusion criteria for the control group were the presence of clinical motor or nonmotor markers for prodromal PD (Berg et al., 2015; Heinzel et al., 2019) or other chronic neurological diseases. The initial design included additional control groups and subgroups and aimed to evaluate several other potential markers, but because of the ongoing pandemic study enrollment was lower than planned; considering the minute sample sizes, these subsidiary data are not discussed.

The clinical evaluation was performed by a neurologist, within a few days from sample collection (typically the day before the blood sample). It included medical history, full neurological examination and assessment of parkinsonism using the modified Hoehn and Yahr scale (Goetz et al., 2004) and the Unified Parkinson’s Disease Rating Scale (UPDRS) part III (Martinez-Martin et al., 1994). Ancillary data were obtained from medical records.

Whole blood samples were collected à jeun on vacutainer clot activator tubes and immediately stored at 4–8°C. Serum was separated and removed within 24 h (typically less than 6) and either fully processed or stored at −20°C. Participants collected their own stool samples (5–10 g) in a sterile plastic container, using the kits they were provided with. Stool samples were collected no later than 3 days after the blood sample (except for the cases of more severe constipation) and kept at room temperature for a maximum of 6 h, then preprocessed using commercially available preparation and extraction tubes (K 6998SAS, K 6999, Immunodiagnostik AG, Germany) and stored at −80°C before being fully processed (up to 8 weeks). The serum and fecal levels of calprotectin and zonulin were determined by enzyme-linked immunosorbent assay (ELISA). For this, we used the commercially available IDK^®^ Calprotectin ELISA K 6927 (stool) and K 6935 (serum) kits and the IDK^®^ Zonulin ELISA K 5600 (stool) and K 5601 (serum) kits (Immunodiagnostik AG, Germany). All samples were processed according to the instruction leaflets that came with the kit.

The results of calprotectin and zonulin levels are expressed as means and standard deviations (SD). Statistical analysis included odds ratio (OR) with 95% confidence intervals (95% CI) and Spearman’s rank correlations coefficient (R). Statistically significant differences were considered at *p*-values < 0.05. For correlations we used IBM^®^ SPSS^®^ Statistics (subscription).

## Results

We fully evaluated 22 patients with sporadic PD (15 males, 7 females) and 16 unmatched healthy controls (9 males, 7 females). Another 4 patients were partially evaluated but could not provide stool samples in due time; their data are not included in the present analysis (see above). The demographic and clinical characteristics of the study population are presented in Table 1. Out of the 22 patients with sporadic PD, 12 fulfilled the MDS diagnostic criteria for clinically established PD (Postuma et al., 2015); the other 10 had the motor onset of PD within the past 5 years and met criteria for clinically probable PD (Postuma et al., 2015); additionally, they fulfilled criteria for clinically established early PD (Berg et al., 2018). Modified Hoehn and Yahr stage ranged from 1 to 4, almost two thirds of the patients having bilateral symptoms and balance impairment [i.e., stage 2.5 or above—see (Goetz et al., 2004)]. Hyposmia (self-reported) was present in 10 PD patients (45.5%). Constipation (self-reported, defined as less than 3 bowel movements per week in the absence of symptomatic treatment) was present in 9 PD patients (41%). Other nonmotor symptoms and concomitant medication are detailed in Supplementary Table 1.

**TABLE 1:** Demographic and clinical characteristics of the study population.

	**Sporadic PD cases** (*n* = 22)	**Healthy controls** (*n* = 16)
Sex	15 (68.18%) males/7 (31.82%) females	9 (56.25%) males/7 (43.75%) females
Age (*p* = 0.0001)	Mean: 68.7 ± 3.51 years, SD 8.4	Mean: 50.5 ± 8.5 years, SD 17.4
BMI (*p* = 0.446)	Mean: 26.7 kg/m^2^ ± 1.77, SD 4.23 BMI 25–29.9 kg/m^2^ (overweight): 8 (36.4%) BMI ≥ 30 kg/m^2^ (obese): 5 (22.7%)	Mean: 25.6 kg/m^2^ ± 2.21, SD 4.51 BMI 25–29.9 kg/m^2^ (overweight): 6 (37.5%) BMI ≥ 30 kg/m^2^ (obese): 3 (18.75%)
Tobacco smoking	Never smokers: 19 (86.4%) Former smokers: 2 (9.1%) Current smokers: 1 (4.5%)	Never smokers: 13 (81.25%) Former smokers: 0 Current smokers: 3 (18.75%)
PD diagnostic (MDS criteria) and motor characteristics	Clinically established PD: 12 (55.5%) Clinically probable PD with criteria for Clinically established early PD: 10 (45.5%) Modified HY stage below 2.5: 8 (36.4%) Modified HY stage 2.5 and above: 14 (63.6%) Mean UPDRS part III: 21.5 ± 5.81, SD 13.9 No. with motor complications: 13 (59.1%)	NA (see exclusion criteria)

**FIGURE 1:**
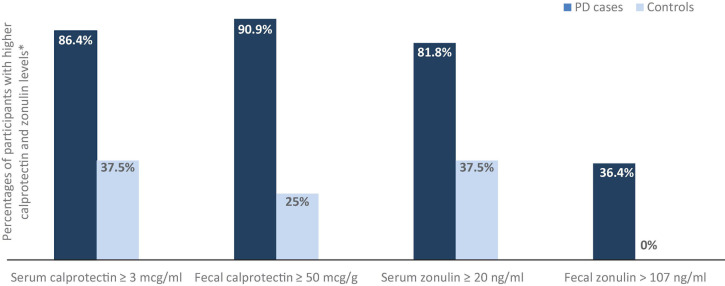
Serum and fecal calprotectin and zonulin levels in the PD group vs. controls. *For serum and fecal calprotectin and fecal zonulin the upper reference limit is used as cut off. Since no study participants had serum zonulin levels above the upper reference limit, but a total of 14 had levels below the lower reference limit (see Table 2), for the purpose of this graph the lower reference limit is used as cut off.

The mean serum calprotectin level was significantly higher in the PD group than in controls (14.26 mcg/ml ± 4.50, SD 10.78 vs. 5.94 mcg/ml ± 3.80, SD 7.75; *p* = 0.0125). Serum calprotectin levels above the upper reference limit were found in samples from 19 out of the 22 PD patients (86.4%) and in 6 (37.5%) out of the 16 controls (OR = 10.56, 95% CI = 2.17–51.42, *p* = 0.0025). The mean fecal calprotectin level was also significantly higher in patients with PD than in controls (164.54 mcg/g ± 54.19, SD 129.68 vs. 56.19 mcg/g ± 35.88, SD 73.22; *p* = 0.0048). Fecal calprotectin levels above the upper reference limit (50 mcg/g) were found in samples from 20 (90.9%) out of the 22 patients and in 4 (25%) out of the 16 controls (OR = 30, 95% CI = 4.75–189.30, *p* = 0.000045); levels above 100 mcg/g were found in samples from 12 (54.5%) PD patients, levels above 150 mcg/g in samples from 8 PD patients (36.4%), above 200 mcg/g in samples from 7 PD patients (31.8%), and above 250 mcg/g in samples from 4 PD patients (18.2%)—with the caveats that 3 out of the 4 patients with the highest levels were treated with levodopa/carbidopa intestinal gel (LGCI) and that all of the patients that were treated with LCGI (*n* = 3) had fecal calprotectin levels above 250 mcg/g; fecal calprotectin exceeded the above cut off values only in 1 (6.25%) of the control samples (exact value: 322.27 mcg/g). Differences between the PD groups and controls remained significant when considering 100 mcg/g as cut off for those age 60 and older and 50 mcg/g for those below 60 (OR = 5.2, 95% CI = 1.15–23.54, *p* = 0.028); this trend also maintained when using 51 mcg/g and 112 mcg/g as age-dependent cut offs (OR = 4.33, 95% CI = 0.96–19.58, *p* = 0.049), as previously done (Mulak et al., 2019). The mean serum zonulin level was higher in the PD group than in controls (26.69 ng/ml ± 3.55, SD 8.51 vs. 19.43 ng/ml ± 2.56, SD 5.22; *p* = 0.0046). No serum zonulin levels above the upper reference limit were found in the study population, but levels below the lower reference limit were identified in 4 (18.2%) out of the 22 PD samples and in 10 (62.5%) of the 16 controls (OR = 0.13, 95% CI = 0.03–0.59, *p* = 0.0068). The mean fecal zonulin level was higher in the PD patient group than in controls (100.19 ng/ml ± 28.25, SD 67.61 vs. 37.30 ng/ml ± 13.26, SD 27.07; *p* = 0.0012). Fecal zonulin levels above the upper reference range limit were found in 8 (36.4%) out of the 22 samples from PD patients and in none of the controls. Levels below the lower reference limit were found only in 2 (12.5%) of the controls. For a summary of these results, see Table 2 and Figure 1.

**TABLE 2:** Calprotectin and zonulin levels.

	**Sporadic PD cases (*n* = 22)**	**Healthy controls (*n* = 16)**	**Significance**
**Serum calprotectin** (reference: <3 mcg/ml)	14.26 mcg/ml ± 4.50, SD 10.78	5.94 mcg/ml ± 3.80, SD 7.75	*p* = 0.0125
Serum calprotectin ≥3 mcg/ml	19 (86.4%)	6 (37.5%)	OR = 10.56, *p* = 0.0025, 95% CI = 2.17–51.42
**Fecal calprotectin** (reference: <50 mcg/g)	164.54 mcg/ml ± 54.19, SD 129.68	56.18 mcg/ml ± 35.88, SD 73.22	*p* = 0.0048
Fecal calprotectin ≥50 mcg/g	20 (90.9%)	4 (25%)	OR = 30, *p* = 0.000045, 95% CI = 4.75–189.30
Fecal calprotectin ≥100 mcg/g	12 (54.5%)	1 (6.25%)	OR = 18, *p* = 0.002, 95% CI = 2.01–161.05
Fecal calprotectin ≥200 mcg/g	8 (36.4%)	1 (6.25%)	OR = 8.57, *p* = 0.034, 95% CI = 0.95–77.57
Fecal calprotectin ≥250 mcg/g	4 (18.2%)	1 (6.25%)	OR = 3.33, *p* = 0.2856, 95% CI = 0.34–33.11
**Serum zonulin** (reference: 20–48 ng/ml)	26.69 ng/ml ± 3.55, SD 8.51	19.43 ng/ml ± 2.56, SD 5.22	*p* = 0.0046
Serum zonulin <20 ng/ml	4 (18.2%)	10 (62.5%)	OR = 0.13, *p* = 0.0068, 95% CI = 0.03–0.59
**Fecal zonulin** (reference: 15–107 ng/ml)	100.19 ng/ml ± 28.25, SD 67.61	37.30 ng/ml ± 13.26, SD 27.07	*p* = 0.0012
Fecal zonulin >107 ng/ml	8 (36.4%)	0	NA
			

In the PD group, we found statistically significant and potentially relevant correlations between serum calprotectin and the modified Hoehn and Yahr stage (*R* = −0.528, *p* = 0.012). Disease duration correlated with parkinsonism severity, assessed with the modified Hoehn and Yahr stage (*R* = 0.775, *p* < 0.001) and the motor UPDRS score/UPDRS part III (*R* = 0.645, *p* = 0.001), as well as with the presence of constipation (*R* = 0.610, *p* = 0.003). Chronic constipation correlated with the motor UPDRS score (*R* = 0.423, *p* = 0.05) and with the daily levodopa equivalent dose (LED) (*R* = 0.43, *p* = 0.46). Motor UPDRS score correlated with female sex (*R* = 0.555, *p* = 0.007). Fecal calprotectin levels above 250 mcg/g correlated with female sex (*R* = 0.457, *p* = 0.042), LCIG treatment (*R* = 0.5, *p* = 0.018) and high serum C reactive protein (CRP) (*R* = 0.677, *p* = 0.011), while levels above 150 mcg/g inversely correlated with self-reported hyposmia (*R* = −0.567, *p* = 0.006); increased fecal calprotectin also correlated with body mass index (BMI) (*R* = 0.499, *p* = 0.018) and obesity (*R* = 0.490, *p* = 0.02). Serum calprotectin levels had negative correlation with the current smoker status (*R* = −0.463, *p* = 0.03). Except for the fecal calprotectin levels above 250 mcg/g, which correlated with female sex in the PD group (*R* = 0.457, *p* = 0.042), we found no significant correlations between serum and fecal calprotectin and zonulin levels or between these and age or sex, neither in patients with PD, nor in controls.

## Discussion

We evaluated the serum and fecal levels of calprotectin and zonulin in people with sporadic PD vs. healthy controls. Despite the small sample size, we found that increased serum and fecal calprotectin levels (i.e., above the upper reference limit) are significantly associated with the risk of PD (OR 10.56 and 30, respectively, *p*-values below 0.005, null hypothesis outside the 95% CIs—see above). The serum zonulin levels were not increased above the upper reference limit in our study, but we found higher mean values in PD compared with controls (*p* = 0.0046); concurrently, levels below the reference range appear to be protective (OR = 0.13, 95% CI = 0.03–0.59, *p* = 0.0068). The mean value of fecal zonulin was higher in PD than in controls (*p* = 0.0012) and values above the upper reference limit were found only in PD (*n* = 8; 36.4%).

Calprotectin is a pleiotropic cytokine-like protein mainly involved in the recruitment of inflammatory cells; it also has bacteriostatic effects that are mediated by zinc-dependent enzymes (Kowalski and Mulak, 2019). It is released or secreted by activated neutrophils, monocytes and endothelial cells, its levels raising rapidly in the presence of bacteria (Diamanti et al., 2010; Jensen et al., 2011; Dhaliwal et al., 2015; Moein et al., 2017; Kowalski and Mulak, 2019). Interestingly, in some experimental settings calprotectin is protective against intestinal injury (Aranda et al., 2018), while in others it may promote amyloidogenesis (Kowalski and Mulak, 2019). Zonulin functions as part of the tight junction system of the mucosal intestinal barrier, modulating its permeability by promoting the disassembly of zona occludens (Ohlsson et al., 2017b). Its secretion or release is mainly triggered by bacteria, but also by some dietary components, such as gluten, higher levels resulting in increased intestinal barrier permeability, which is found along inflammatory changes in people with PD and other neurodegenerative conditions (Ohlsson et al., 2017b; Kowalski and Mulak, 2019).

Fecal calprotectin is a well-established biomarker of intestinal inflammation, routinely used in the diagnostic and monitoring of IBD. However, it is not specific for IBD. Other circumstances that may increase fecal calprotectin levels include the use of certain drugs, especially NSAIDs and possibly PPIs, and certain disorders, such as infectious enterocolitis, celiac disease and colorectal cancer (Khaki-Khatibi et al., 2020). In people with chronic diarrhea and other symptoms suggestive of IBD, fecal calprotectin below 50 mcg/g excludes clinically relevant intestinal inflammation, while levels above 250 mcg/g are highly specific (Diamanti et al., 2010; Jensen et al., 2011; Dhaliwal et al., 2015; Moein et al., 2017). Noteworthy, IBD is a risk factor for developing PD (Berg et al., 2015; Camacho-Soto et al., 2018a; Heinzel et al., 2019; Park et al., 2019; Villumsen et al., 2019; Weimers et al., 2019; Zhu et al., 2019; Lee et al., 2021) and a promising candidate risk marker for diagnosing prodromal PD (Heinzel et al., 2019). The role of serum or plasma calprotectin in the diagnostic and follow-up of chronic inflammation is not so well defined (Wang and Liang, 2019).

Interestingly, most people with sporadic PD develop gastrointestinal symptoms related to decreased transit time, constipation preceding the motor dysfunction with more than a decade in some cases (Postuma et al., 2015). Putatively, these symptoms are related to PD pathology in the enteric nervous system (Braak et al., 2006; Devos et al., 2013). Gut dysbiosis is common in PD and can result in local inflammatory changes and barrier dysfunction/disruption, which may increase alpha-synuclein expression and facilitate its exposure to amyloidogenic compounds found in the gut, thus possibly contributing to key pathogenic events in PD; prospective evidence is nevertheless scarce (Stolzenberg et al., 2017; Nuzum et al., 2020; Vascellari et al., 2021). The coexistence of an amyloidogenic gut microbiota with intestinal inflammation (leading to local overexpression of alpha-synuclein) and altered intestinal barrier permeability (exposing alpha-synuclein to the amyloidogenic xenobiotics) may play a key role in triggering the initial alpha-synuclein conformational changes in some people with sporadic PD (Sampson et al., 2020). Disturbances in the microbiota-gut-brain axis may also contribute to neurodegeneration, interfering with neuronal susceptibility to stressors and ultimately with neuronal survival (Devos et al., 2013; Stolzenberg et al., 2017; Nuzum et al., 2020; Vascellari et al., 2021).

In our study higher levels of calprotectin correlated with milder parkinsonism on the modified Hoehn and Yahr scale (*p* = 0.012), which would suggest that the intestinal inflammation is higher in the earlier stages of the disease. We also found that increased calprotectin levels (above 150 mcg/g) correlated negatively with self-reported hyposmia (*p* = 0.006); this cannot be interpreted solely based on our study, but fits with the hypothesis of a more heterogenous development of sporadic PD (Rietdijk et al., 2017), the initial pathogenic events being either intestinally-centered, or olfactory-centered, or both.

The results of our study are in line with those of two other previous studies that compared fecal calprotectin and zonulin levels in 35 PD patients and 20 controls, and in 34 PD patients and 28 age-matched controls, respectively (Schwiertz et al., 2018; Mulak et al., 2019). Both studies found significant associations between higher fecal calprotectin and PD, while only the latter found a significant association between higher fecal zonulin and PD. In addition to these studies, we also evaluated serum levels of calprotectin and zonulin and found significant associations between higher levels and PD. Although in people with IBD, serum calprotectin correlates with the fecal levels (Kalla et al., 2016), we found no such correlation. The present study poses a series of limitations that mandate caution when interpreting its results. Most important, the number of participants in each group was lower than planned and did not allow matching, therefore our results warrant confirmation on larger groups that are matched for age, sex and dietary habits (the latter being a possible confounding factor, especially in respect to zonulin levels). Except for the highest fecal calprotectin levels being more common in females with PD, we found no significant correlations between serum and fecal calprotectin and zonulin levels and age or sex. Fecal calprotectin levels may increase with age, one study reporting median values of 18 mcg/g in healthy adults below 60 years old and 27 mcg/g in older individuals (Khaki-Khatibi et al., 2020). In our study, the association between increased fecal calprotectin and PD remained significant even when using age-dependent cut off values. As expected based on the available literature (Sapone et al., 2006; Khaki-Khatibi et al., 2020; Seethaler et al., 2021), we found no significant correlation between zonulin levels and age or sex. Dietary patterns were not assessed in our study, however, serum zonulin levels may correlate with macro- and micronutrient intake, including the total carbohydrate and vitamin D, respectively (Morkl et al., 2018). Increased BMI is another potential confounding factor, both for calprotectin and zonulin (Mortensen et al., 2009; Ohlsson et al., 2017a; Grand et al., 2020). In our study there were no significant differences in BMI between the PD group and controls (*p* = 0.446); in agreement with the available literature (Mortensen et al., 2009; Grand et al., 2020), increased calprotectin levels correlated with increased BMI and obesity in the PD group. All the PD patients included in our study had symptomatic treatments with dopamine agonists or levodopa-based drugs, which are possible confounding factors. We found no correlations between these drugs, LED, and calprotectin or zonulin levels, but this could be related to the small sample size; notably, very high fecal calprotectin levels correlated with LCIG (*p* = 0.018). Another limitation of our study is that serum and fecal calprotectin and zonulin levels were not confirmed on a different occasion or by further gastroenterological evaluation within the study, meaning that the results could reflect isolated circumstances (that were not accounted for by the exclusion criteria) rather than a chronic ongoing process related to PD.

Concluding, our findings suggest an association between sporadic PD and serum and fecal markers of intestinal inflammation and permeability. This is consistent with data from two other small studies and underlines the importance of further investigating the gastrointestinal tract to better understand the pathogenic events involved in the initiation and progression of sporadic PD. Provided future research confirms the relation between increased calprotectin levels and PD, serum and fecal calprotectin testing could help improve the accuracy of current clinical diagnostic criteria.

## Data Availability Statement

The raw data supporting the conclusions of this article will be made available by the authors, without undue reservation.

## Ethics Statement

The protocol of the study was approved by the local Ethics Committee of Colentina Clinical Hospital (EMI-BPs, 3/16.04.2019). The patients/participants provided their written informed consent to participate in this study.

## Author Contributions

LD drafted and revised the manuscript; she also designed and drafted the protocol of the study (as part of her Ph.D., under the supervision of BOP), critically revised the laboratory protocol used in the study, contributed to the ethics submission, to the recruitment and evaluation of some study participants and to data collection, and processing. DM performed the laboratory tests and revised the manuscript; she also drafted the laboratory protocol used in the study, critically revised the protocol of the study and contributed to the recruitment of study participants. AD critically revised the protocol of the study, the laboratory protocol and the ethics submission, contributed to the recruitment and evaluation of study participants and to data collection and processing, and revised the manuscript. AL critically revised the ethics submission, contributed to the recruitment and evaluation of study participants, data collection and processing, and revised the manuscript. DT and LC contributed to the recruitment and evaluation of study participants and revised the manuscript. EM, MG, and LC critically revised the laboratory protocol used in the study and the protocol of the study and revised the manuscript. BOP coordinated and critically revised all aspects related to the study and the manuscript, was responsible for PN 19.29.02.01, granting funding for this work, was the PI of the study, and the supervisor of LD’s Ph.D. All authors approved the final version of the manuscript.

## Conflict of Interest

The authors declare that the research was conducted in the absence of any commercial or financial relationships that could be construed as a potential conflict of interest.
