# Supplementary material for: Serum and Fecal Markers of Intestinal Inflammation and Intestinal Barrier Permeability Are Elevated in Parkinson’s Disease
Source: Front Neurosci. 2021 Jun 18;15:689723. doi: 10.3389/fnins.2021.689723 (PMC8249847; doi:10.3389/fnins.2021.689723)
Supplement: Supplementary file 1 [file Table_1.docx]

Supplementary Table 1. Additional clinical characteristics

| **Sporadic PD cases** | |
| --- | --- |
| Age at onset (mean) | 61.8 ± 4.1 years, SD 9.7 |
| PD disease duration - since motor onset | Mean disease duration: 7 ± 2.4 years, SD 5.7  No. of patients with PD for less than 5 years: 10 (45.5%) |
| Parkinsonism subtype | Akinetic-rigid subtype: 10 (45.5%)  Tremor-dominant subtype: 11 (50%)  Mixed subtype: 1 (4.5%) |
| PD nonmotor symptoms / manifestations | Hyposmia (self-reported): 10 (45.5%)  Constipation* (self-reported): 9 (41%)  Orthostatic hypotension (assessed): 5 (22.7%)  Neurocognitive impairment (assessed): 5 (22.7%)  Sleep disorders (medical records): 6 (27.3%)  Depression (medical records): 9 (40.9%) |
| Chronic gastrointestinal symptoms (other than constipation) | Dysphagia: 2 (9.1%)  Diarrhea: 1 (4.5%)  Meteorism: 10 (45.5%) |
| Other chronic diseases  (except PD) | Cardiovascular disorders: 10 (45.5%)  Hypothyroidism: 1 (4.5%)  Others: 4 (18.2%)  None: 8 (36.4%) |
| Concomitant medication / therapies | Mean daily LED: 830.5 mg  No. treated with levodopa: 20 (90.9%)  No. treated entacapone: 5 (22.7%)  No. treated with dopamine agonists: 2 (9.1%)  No treated with rasagiline: 17 (77.3%)  No. treated with LCGI: 3 (13.6%)  No. with deep brain stimulation (DBS): 0  No. on rivastigmine (transdermal): 2 (9.1%)  No. on memantine: 1 (4.5%)  No. on low dose aspirin: 3 (13.6%)  No. on other drugs (one or more): 8 (36.4%) |

* defined as less than 3 bowel movements per week, in the absence of symptomatic treatment, for more than 3 months.

NB. The controls had no concomitant PD nonmotor symptoms or chronic gastrointestinal symptoms. They had no chronic diseases, except for cardiovascular disorders (n = 3, 18.75%), and no potentially relevant concomitant medication, except for low dose aspirin and antihypertensive drugs (n = 3, 18.75%).
